# Supplementary material for: Netcombin: An algorithm for constructing optimal phylogenetic network from rooted triplets
Source: PLoS One. 2020 Sep 18;15(9):e0227842. doi: 10.1371/journal.pone.0227842 (PMC7500971; doi:10.1371/journal.pone.0227842)
Supplement: S4 File — (ZIP) [file pone.0227842.s004.zip › SequenceData-Triplet/sequenceData-10-20-30-0-Triplet/length 500, numseq 40, rate 10^-7/5 seq.pdf]

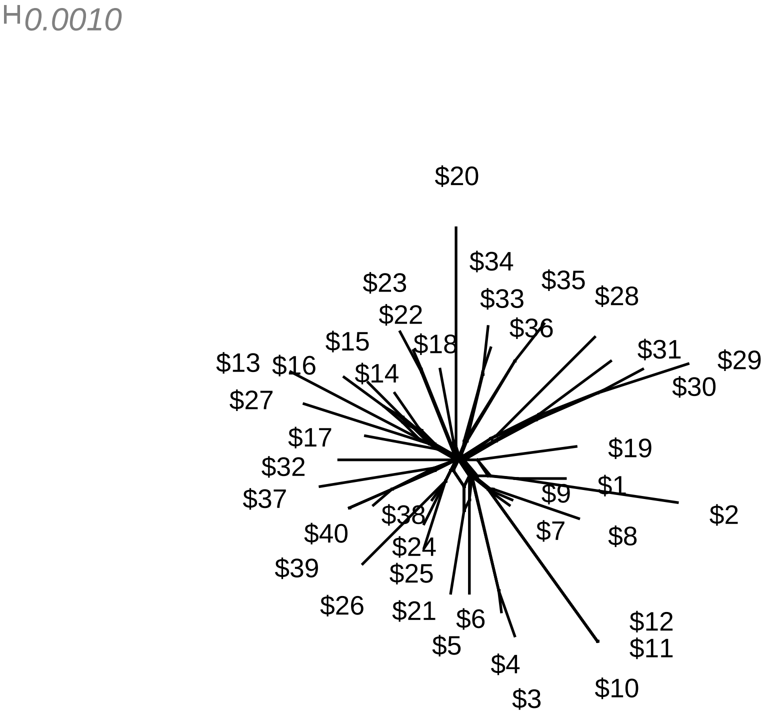
0.0010
$20
$34
$23
$35
$33
$28
$22
$36
$15
$18
$31
$29
$13
$16
$14
$30
$27
$17
$19
$32
$1
$9
$37
$2
$38
$7
$8
$40
$24
$39
$25
$26
$21
$12
$6
$5
$11
$4
$10
$3
